# MARKED DIFFERENCES IN LOCAL BONE REMODELLING IN RESPONSE TO DIFFERENT MARROW STIMULATION TECHNIQUES IN A LARGE ANIMAL

**DOI:** 10.22203/eCM.v041a35

**Published:** 2021-05-19

**Authors:** H.M. Zlotnick, R.C. Locke, B.D. Stoeckl, J.M. Patel, S. Gupta, K.D. Browne, J. Koh, J.L. Carey, R.L. Mauck

**Affiliations:** 1 McKay Orthopaedic Research Laboratory, Department of Orthopaedic Surgery, University of Pennsylvania, Philadelphia, PA, USA; 2 Department of Bioengineering, University of Pennsylvania, Philadelphia, PA, USA; 3 Translational Musculoskeletal Research Center, CMC VA Medical Center, Philadelphia, PA, USA; 4 Department of Orthopaedics, Emory University, Atlanta, GA, USA; 5 Center for Neurotrauma, Neurodegeneration and Restoration, CMC VA Medical Centre, Philadelphia, PA, USA; 6 Orthopaedic and Spine Institute, NorthShore University Health System, Evanston, IL, USA

**Keywords:** Bone remodelling, fluorochrome, large-animal models, microfracture, marrow stimulation, drilling

## Abstract

Marrow stimulation, including subchondral drilling and microfracture, is the most commonly performed cartilage repair strategy, whereby the subchondral bone plate is perforated to release marrow-derived cells into a cartilage defect to initiate repair. Novel scaffolds and therapeutics are being designed to enhance and extend the positive short-term outcomes of this marrow stimulation. However, the translation of these newer treatments is hindered by bony abnormalities, including bone resorption, intralesional osteophytes, and bone cysts, that can arise after marrow stimulation. In this study, three different marrow stimulation approaches – microfracture, subchondral drilling and needle-puncture – were evaluated in a translationally relevant large-animal model, the Yucatan minipig. The objective of the study was to determine which method of marrow access (malleted awl, drilled Kirschner wire or spring-loaded needle) best preserved the underlying subchondral bone. Fluorochrome labels were injected at the time of surgery and 2 weeks post-surgery to capture bone remodelling over the first 4 weeks. Comprehensive outcome measures included cartilage indentation testing, histological grading, microcomputed tomography and fluorochrome imaging. Findings indicated that needle-puncture devices best preserved the underlying subchondral bone relative to other marrow access approaches. This may relate to the degree of bony compaction occurring with marrow access, as the Kirschner wire approach, which consolidated bone the most, induced the most significant bone damage with marrow stimulation. This study provided basic scientific evidence in support of updated marrow stimulation techniques for preclinical and clinical practice.

## Introduction

Focal cartilage injuries are common, impacting ~ 1 million Americans annually (Mithoefer et al., 2009). Due to the poor intrinsic healing capability of articular cartilage, such lesions may progress to degenerative OA if left untreated (Heijink et al., 2012). In the United States alone, OA impacts over 27 million people, resulting in over 100 billion US dollars of healthcare costs (Jafarzadeh and Felson, 2018; Murphy and Helmick, 2012). With severe OA, joint arthroplasty may be the only option to restore mobility and improve life quality.

In an effort to repair cartilage defects, and ultimately prevent joint-wide OA, the most common procedure is MST (Martín et al., 2019). MST is a relatively simple and cost-effective strategy to introduce regenerative cells into a cartilage defect. The process involves the initial debridement of the cartilage defect, followed by perforation of the subchondral bone plate using either a fluted drill bit, K-wire (subchondral drilling) or surgical awl (microfracture) (Gao et al., 2018; Steadman et al., 2002). Then, the pressure within the marrow compartment promotes marrow-derived fluid and cells to populate the cartilage defect, form a clot and serve as a template for the formation of a fibrous matrix (DiBartola et al., 2016).

While MST primarily leads to fibrous tissue formation (Knutsen et al., 2004), it remains an important first-line treatment for cartilage defect repair. MST can be performed as a single arthroscopic procedure and has been shown to improve knee function in 70-95 % of patients (assessed 2-11 years post-surgery) (Gobbi et al., 2005; Knutsen et al., 2007; Steadman et al., 2003). To further improve and extend these outcomes, MST has been combined with a collagen type I/III membrane to engage and hold the marrow clot in place (Volz et al., 2017). This procedure is referred to as AMIC and has shown favourable results in comparison to MST alone 5 years post-surgery. In addition to AMIC, several laboratories have developed technologies focused on augmenting MST to direct cell differentiation and produce hyaline-like cartilage (Kim et al., 2015; Madry et al., 2020; Miller et al., 2014; Morisset et al., 2007; Patel et al., 2020; Sennett et al., 2021; Zanotto et al., 2019). Only a few of these strategies have progressed beyond the preclinical testing stage (Sharma et al., 2013).

Given the role of the subchondral bone in OA, it is likely that for augmented MST to reach its full potential, steps must be taken to address unintentional bony abnormalities that may arise from marrow access. This includes reports of bone resorption, intralesional osteophytes and the formation of bone cysts (Cole et al., 2011; Zedde et al., 2016). These subchondral irregularities likely impede the translation of novel scaffolds and therapeutics (Sennett et al., 2021) and may also limit revision treatment options in the case of MST failure (Riff et al., 2020). Intuitively, without a stable foundation on which to form, the repair tissue may be predisposed to failure. To reduce bone damage, it may be beneficial to scale down tool sizing. Indeed, both small (1 mm) diameter awls (Orth et al., 2016) and small (1 mm) diameter K-wires have recently been shown to improve MST outcomes in comparison to larger instrumentation (Eldracher et al., 2014). Others have suggested that small diameter (1 mm) holes that are deeper (9 mm depth) may preserve the underlying bone in comparison to more traditionally larger (2 mm) and shallower (2-4 mm) holes that can fragment and compact the bone (Chen et al., 2009; Zedde et al., 2016).

While there is growing evidence to support the use of smaller diameter and deeper access for MST, the mechanism for creating these narrow, extended holes (whether it be by impaction, drilling or another method) is not yet determined. To date, only 7 studies have directly compared the standard MST approaches – microfracture and subchondral drilling – and only 3 of these studies included multiple outcome measures (Kraeutler et al., 2020). Thus, there is a lack of knowledge regarding how the method of hole creation impacts the subchondral bone. Therefore, the goal of this study was to evaluate the impact of 3 different methods of MST (awl-based microfracture, needle puncture and drilling) on the underlying subchondral bone in a Yucatan minipig model of cartilage defect repair. The needle puncture device (SmartShot^®^ marrow access device, Marrow Access Technologies, Minnetonka, MN, USA) employed is spring-powered and self-retracting and, therefore, different from the awl in the consistency and speed of entry and exit from the bone and its overall penetration depth into the bone (~ 4× deeper than an awl). For this study, one SmartShot^®^ device size-matched in diameter to the microfracture awl and another SmartShot^®^ device size-matched in diameter to the K-wire were included, resulting in 4 experimental treatment groups for comparison. The study hypothesis was that the smaller SmartShot^®^ device (0.9 mm diameter) would cause the least bone resorption and compaction, as assessed 4 weeks post-MST. Also, bone fluorochrome labelling was implemented to track bone remodelling over the study duration. This technique was first pioneered by Dr Berton Rahn and Dr Stephen Perren (Rahn and Perren, 1971) and provided a unique window into the dynamics of bone healing at these MST marrow access sites.

## Materials and Methods

### Animal study

**Fig 1.**
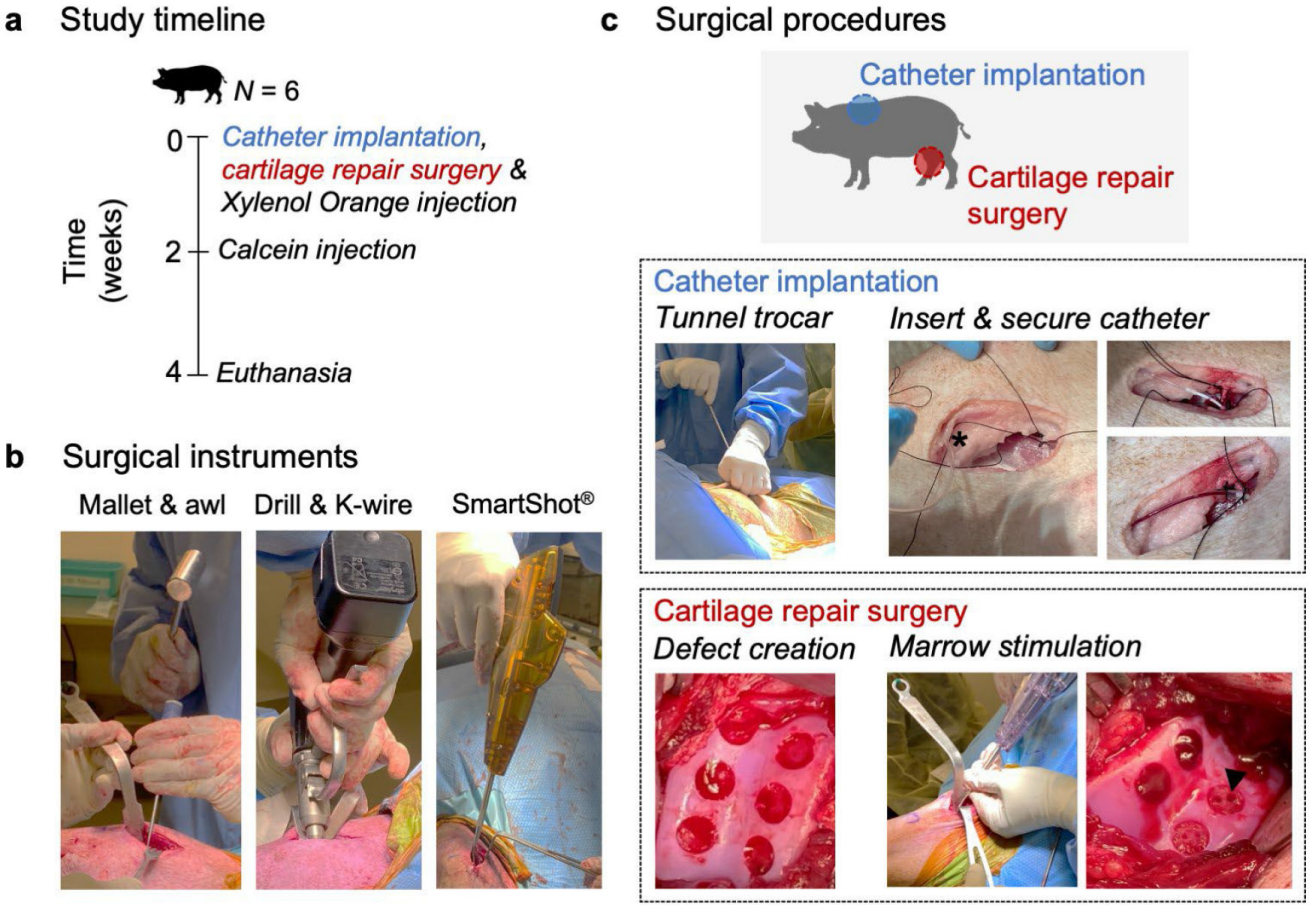
Study overview. (**a**) Study timeline. (**b**) Surgical instruments for cartilage repair procedure. (**c**) Surgical procedures. Catheter implantation: an indwelling catheter was implanted in the brachiocephalic vein for fluorochrome delivery. * denotes tunnelled catheter. Cartilage repair surgery: six full-thickness chondral defects (5 mm diameter) were created in the trochlea of the stifle joint. Arrowhead: MST hole.

All animal procedures were approved by the IACUC at the University of Pennsylvania. 6 skeletally mature (age 12 months at the beginning of the study) male Yucatan minipigs (Sinclair Bioresources, Auxvasse, MO, USA) were used. Under a single anaesthetic induction, two separate surgical procedures were performed (Fig. 1). Both the left upper extremity and right stifle joint were sterilely draped and prepared using betadine. In the first procedure, an indwelling catheter was implanted in the brachiocephalic vein. In a second procedure, a unilateral stifle joint surgery was performed using a minimally invasive open approach (Bonadio et al., 2017). During the stifle joint surgery, a bone fluorochrome label was infused through the implanted catheter. 2 weeks post-surgery, a second bone fluorochrome label was infused to track bone remodelling. Animals were euthanised 4 weeks post-surgery. Cartilage indentation testing, μCT (bone resorption), mineralised cryohistology (fluorochrome labelling, tartrate resistant phosphatase staining) and demineralised paraffin-wax histology (SafO/FG and haematoxylin and eosin stains) with blinded ICRS II scoring were performed on the defects from the operative limbs post-euthanasia. Four of the non-operative limbs were used for a time 0 assessment of bone compaction by μCT.

#### Catheter implantation

For the catheter implantation, a 4 cm incision was made in the left neck 4.5 cm lateral to the manubrium. Dissection was carried down to the brachiocephalic vein. Medial and lateral ties (0 silk) were loosely tied to the vein. A cannula and a trocar were driven subcutaneously from the neck incision to the left scapula. The trocar was removed and the catheter (79 cm length, Luer lock, SAI Infusion Technologies, Lake Villa, IL, USA, CPJC-12) was passed through the cannula, leaving the Luer hub exteriorised at the left scapula. An 11-blade was used to create an opening in the brachiocephalic vein for passage of the catheter. The silk ties were fastened to secure the catheter to the vein. Both the neck and scapular incisions were closed and the catheter was flushed to assure its function.

#### Stifle joint surgery

Immediately after closing the incisions from the catheter implantation, a unilateral stifle joint procedure was performed on the right hind limb. A medial parapatellar skin incision exposed the trochlea. Six full-thickness chondral defects were created in the trochlear groove using a 5 mm biopsy punch. A curette was used to excise cartilage tissue from within the bounds of the scored defect. Care was taken to preserve the subchondral plate. In each animal, a single defect was left untreated (empty) as a control (*n* = 6). Treatment groups included: awl-based microfracture (diameter: 0.8 mm, depth: 2 mm), SmartShot^®^ (diameter: 0.9 mm, depth: 8 mm), SmartShot^®^ (diameter: 1.2 mm, depth: 8 mm) and drilling with a K-wire (diameter: 1.25 mm, depth: 6 mm). For each treatment, 3 MST holes were created, except for the SmartShot^®^ (1.2 mm) where the outer housing limited the hole number to 2. The 4 treatment groups were positionally randomised across 28 defects (*n* = 7/treatment group). All animals received at least 1 of each treatment group. 2 defects were used for another study (36 total defects across the 6 animals). Sample size was determined based on an *a priori* power analysis using previous data in this animal model (Pfeifer et al., 2017).

#### Fluorochrome injections and catheter maintenance

Xylenol orange (90 mg/kg, Millipore, 398197) was injected slowly over 1 h through the indwelling catheter during the stifle joint procedure (Funk et al., 2009). Xylenol orange was dissolved in 100 mL saline (Medline, Wilmer, TX, USA, EMZ111240) and sterile filtered. 2 weeks post-surgery, the animals were sedated, anaesthetised and intubated. Under anaesthesia, calcein (15 mg/kg, Millipore, C0875) was injected slowly over 1 h through the indwelling catheter (Funk et al., 2009). Calcein was dissolved in 100 mL of 1.4 % (wt/vol) sodium bicarbonate (Millipore, S5761) and sterile filtered. Blood draws were performed through the catheter before each fluorochrome injection, 1 min post-injection, 1 d post-injection and 1 week post-injection. Blood samples were analysed to measure ionised calcium (Antech Diagnostics, New Hyde Park, NY, USA). The catheters were flushed twice daily with saline and heparin locked (100 U/mL, Medsupply Partners, Atlanta, GA, USA, BD-306423) to maintain patency. One animal’s catheter was not patent at the 1 d time point after the calcein injection, decreasing the sample size to 5 animals for the 1 d and 1 week time points. All animals wore Thundershirts for dogs (Thunderworks, Durham, NC, USA, BSSXXL-T01) to protect the exteriorised catheter hub and prevent pull-out.

#### Euthanasia and cadaveric use of non-operative stifle joint

All animals were euthanised 4 weeks post-surgery. The operative limbs were prepared for cartilage indentation testing. 4 of the non-operative contralateral stifle joints (left hind limbs) were used to assess initial (time 0) local bone compaction surrounding the MST holes. For this assessment, mock surgeries were performed on the cadaveric joints, mimicking the operative limbs. Briefly, chondral defects (*n* = 6 defects/knee) were created using a 5 mm biopsy punch and curette. Each limb had one non-treated (empty) defect control (*n* = 4 empty defects) and the surgical treatments [microfracture (0.8 mm), SmartShot^®^ (0.9 mm), SmartShot^®^ (1.2 mm), K-wire (1.25 mm)] were randomised to the remaining defects (*n* = 5 defects/treatment).

### Cartilage indentation testing

Following euthanasia, a hand saw was used to produce 1 cm^3^ osteochondral units containing the chondral defect from the operative limbs (Sennett et al., 2021). Control cartilage was collected from the distal end of the trochlea. Each sample (*n* = 6-7 defects/group) was potted in PMMA (Ortho-Jet, Lang Dental, Wheeling, IL, USA) to secure the bone. All samples were tested. Samples were covered in PBS with protease inhibitors (Roche Complete, Millipore) and tested within 5 h of collection. Each sample was placed in a custom rig equipped with an XY positioning stage and a goniometer to ensure that the cartilage surface was indented perpendicular to the 2 mm diameter spherical indenter (Meloni et al., 2017). A load of 0.1 N was applied at an initial rate of 0.1 mm/s and held over 900 s as the tissue underwent creep displacement. The compressive modulus (E_y−_) was computed by fitting the creep data to a Hertzian Biphasic analytical model (Moore et al., 2016). Cartilage thickness was experimentally calculated from samples excised during surgery and input into the analytical model.

### μCT

After indentation testing, all samples (*n* = 7 defects/group) were removed from the PMMA, submerged in PBS with protease inhibitors and imaged by μCT (Scanco μCT50, Scanco Medical). Scans were conducted utilising the following parameters: 1,500 projections, 851 ms per 1 exposures/projection, voltage 70 kVp, current 85 μA, isotropic voxel size 10.3 μm. The volume of bone resorption was calculated using the Scanco evaluation software by manually contouring the void space underneath the neocartilage. The MST holes were excluded from these measurements.

Parallel μCT scans were performed on the osteochondral units from the cadaveric time 0 surgeries using the contralateral hind limbs. The same μCT imaging parameters were used for these samples. For each treatment condition, the local BV/TV surrounding individual MST holes (*n* = 6 holes analysed/treatment) was calculated. Using the Scanco evaluation software, an outer circle was drawn with a diameter 0.65 mm longer than the original hole diameter. This value was selected to ensure that the volume measured was unique to that hole and not overlapping with another MST hole within the same defect. Subsequently, the perimeter of the MST hole was contoured within this outer circle, creating a hollow cylinder. The BV/TV was computed for this hollow cylinder, excluding the inner void space.

### Histology

#### Mineralised cryohistology

Samples were fixed immediately after μCT scanning for 24 h in 10 % neutral buffered formalin. After fixing, samples were infiltrated for 48 h with a 10 % sucrose (Fisher Scientific, S25590) and 2 % polyvinylpyrrolidone (Millipore, P5288) solution. Then, samples were embedded in OCT compound and sectioned (18 μm/section) undecalcified using cryofilm until reaching the midplane of the defect using a motorised cryostat (CM1950, Leica) (Dyment et al., 2016). Tape-stabilised, frozen sections were subjected to two rounds of imaging using a Zeiss Axio Scan.Z1 digital slide scanner, including imaging of (i) fluorochrome labels and dark field, (ii) TRAP staining with TO-PRO-3 Iodide (Scientific, T3605) counterstain. For TRAP staining, sections were incubated for 1 h in TRAP buffer (0.92 % sodium acetate anhydrous, 1.14 % L_(+)_-tartaric acid, 1 % glacial acetic acid pH 4.1-4.3) and then incubated for 1 h under ultraviolet light with ELF97 substrate (Thermo Fisher Scientific, E6588) in TRAP buffer.

Fluorochrome labels (% area) were quantified in the region surrounding the MST holes. Images were split into red and green channels, representing the xylenol orange and calcein signals, respectively. Then, images were binarised and rotated in Fiji to orient the MST holes perpendicular to the surface (Schindelin et al., 2012). A region of interest (3 mm by 1 mm) was defined immediately adjacent to the MST hole. Sample size (*n* = 6-7 holes/treatment) was determined by the number of distinct sections with an apparent MST hole. Only one hole was measured for a given defect. TRAP staining (*n* = 5-6 defects/treatment) was quantified by measuring the number of positive TRAP pixels within a 7 mm by 7 mm area normalised by the bone area within this region.

#### Demineralised paraffin-wax histology

After cryosectioning one half of each defect, all samples (*n* = 7 defects/group) were removed from OCT and decalcified (Formical 2000, StatLab Medical Products) for 2 d. Then, these decalcified samples were processed for paraffin-wax histology. Sections from the midplane of each defect were cut (7 μm) using a paraffin microtome (RM2245, Leica) and stained with haematoxylin and eosin and SafO/FG.

#### Histological scoring

SafO/FG-stained sections were assessed using the modified ICRS II scoring system (Mainil-Varlet et al., 2010). The following parameters were scored on a continuous scale from 0 (poor defect repair) to 100 (normal articular cartilage): defect fill, integration to surrounding cartilage, matrix staining, surface architecture, basal integration, subchondral bone abnormality, vascularisation, surface/superficial assessment, mid/deep zone assessment, overall assessment. Three blinded reviewers with expertise in cartilage histomorphology scored each sample. Scores for each defect were averaged and plotted by treatment group.

### Data analysis

All quantitative data were analysed using GraphPad Prism (version 8.4.3 for MacOS, GraphPad Software). For mechanical testing, histological scoring, blood analyses, fluorochrome labelling, bone resorption and TRAP data, a one-way ANOVA was performed with *post-hoc* Tukey’s corrections for multiple comparisons. For the bone compaction analysis, a Kruskal-Wallis test was performed with a *post-hoc* Dunn’s test. All plots were made in Prism.

## Results

### Surgical outcomes and blood analyses

**Fig 2.**
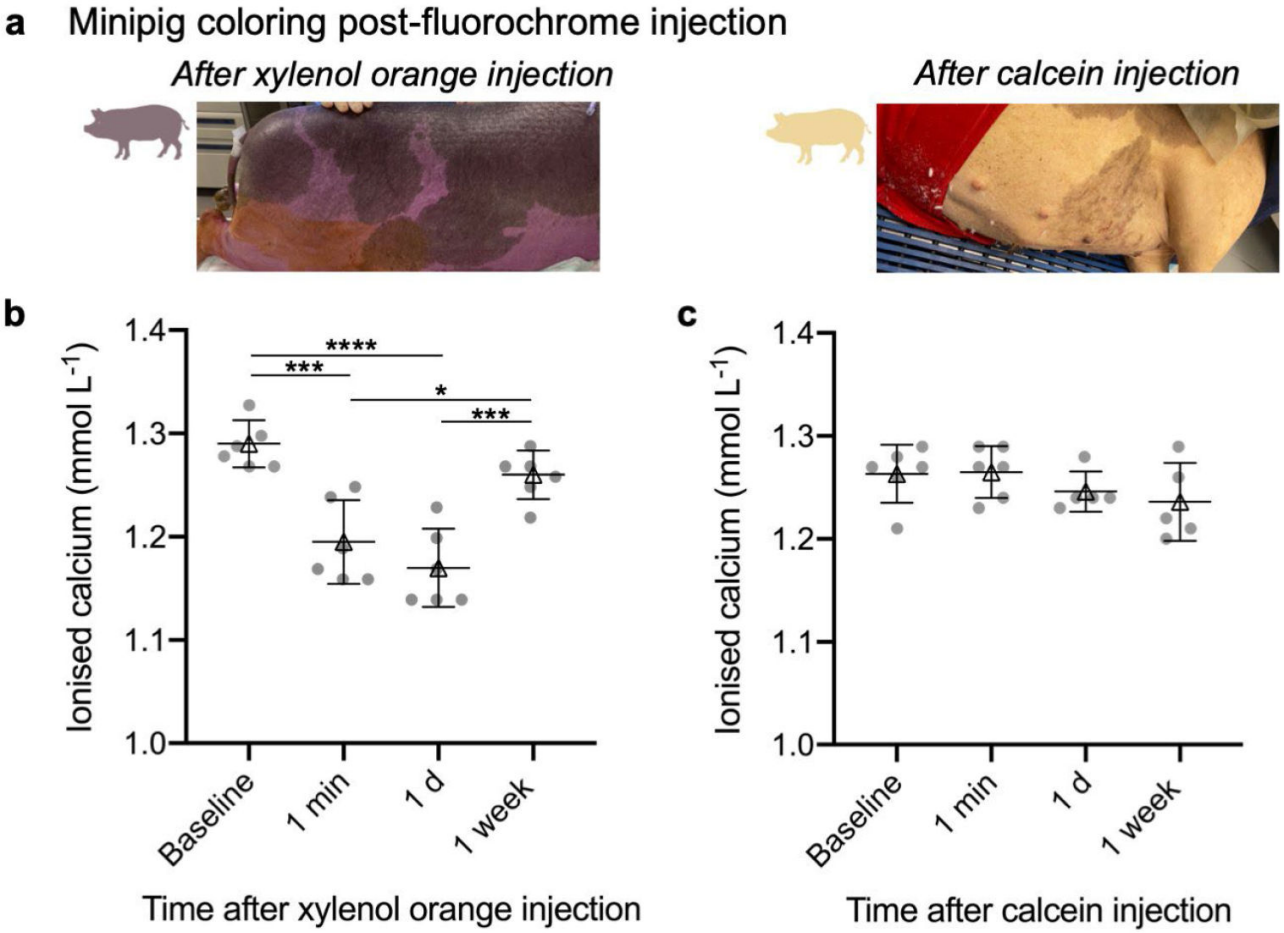
Blood analyses before and after injecting fluorochrome labels. (**a**) Minipig skin colouring post-fluorochrome injection. Immediately after xylenol orange injection, the animals turned purple. The animals turned slightly yellow after calcein injection. Animal colouring returned to normal within 3 h post-injection. (**b,c**) Ionised calcium levels in the blood after (**b**) xylenol orange and (**c**) calcein injection through 2 weeks post-surgery. For both plots, individual animal data are represented by the circles. Triangles and error bars represent the mean ± standard deviation. * *p* < 0.05, *** *p* < 0.001, **** *p* < 0.0001. *N* = 5-6 animals.

All animal procedures were performed without complications (Fig. 1) and animals were allowed weight bearing within 2 h post-surgery. Animal skin coloration changed dramatically immediately after fluorochrome injection (Fig. 2a), evidence of the systemic distribution of the label. The xylenol orange and calcein injections caused the animals' skin to transiently turn purple and yellow, respectively. Animal skin coloration returned to normal within few hours post-injection. Blood samples were taken both pre- and post-injection (1 min, 1 d, 1 week) and analysed for ionised calcium levels. There was a slight decrease in ionised calcium after the injection of xylenol orange label (Fig. 2b) but this returned to baseline within 1 week post-injection. After the calcein injection, there were no changes in ionised calcium (Fig. 2c).

### Mechanical testing and histology of regenerating cartilage

**Fig 3.**
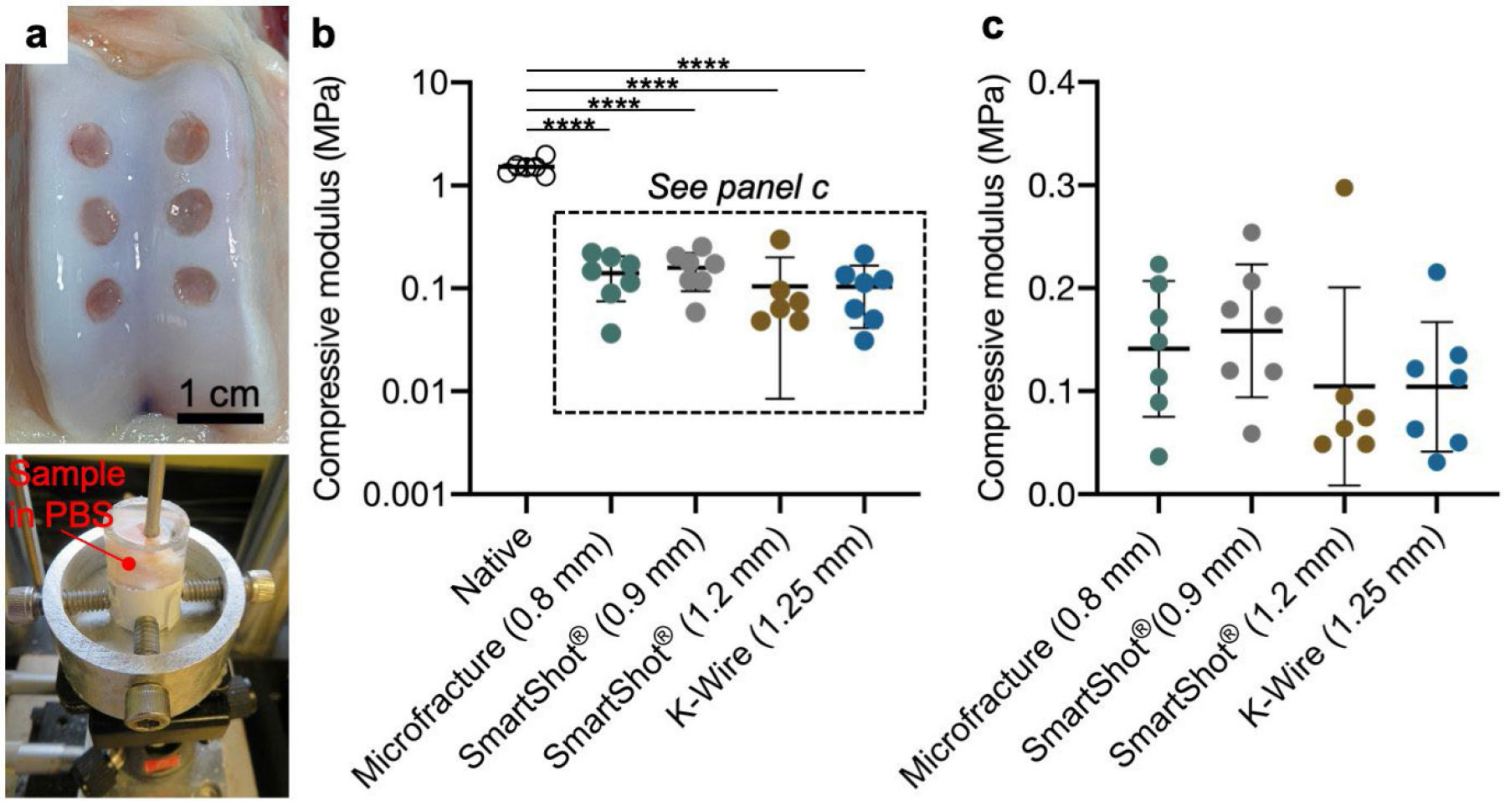
Macroscopic cartilage repair and cartilage mechanical properties. (**a**) Macroscopic image of joint 4 weeks post-surgery and indentation testing setup. (**b**) Compressive modulus (displayed on Log_10_ scale) including the native cartilage controls. **** *p* < 0.0001. (**c**) Compressive modulus (linear scale, from **b**). All plots show mean ± standard deviation. *n* = 7 defects/treatment. *N* = 6 animals.

**Fig 4.**
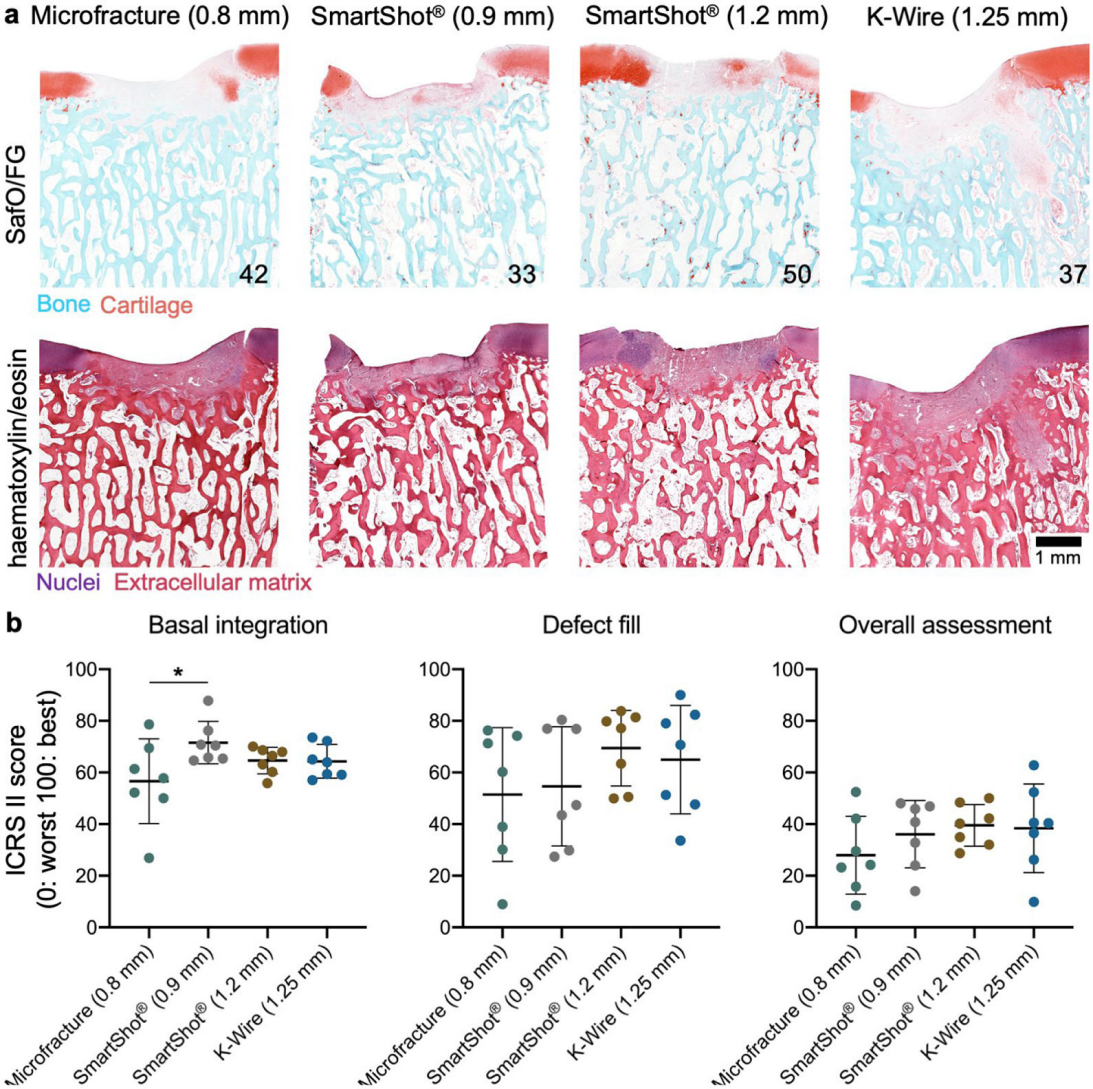
Cartilage repair quality 4 weeks after MST. (**a**) Histological staining (SafO/FG and haematoxylin/eosin) of representative defects. Numbers represent the overall ICRS II histological score for that specimen. Limited proteoglycan deposition was observed in the defects at this early time point. (**b**) Blinded ICRS II histological scoring (0: worst, 100: best). * *p* < 0.05. Bars indicate mean ± standard deviation. *n* = 7 defects/treatment. *N* = 6 animals.

Indentation testing was performed to assess the mechanical properties of the repair tissue (Fig. 3a). Native cartilage controls were taken from the distal portion of the operative femoral trochlea. As expected, all treatment groups had a significantly lower compressive modulus in comparison to the native cartilage controls (Fig. 3b). There were no differences observed between MST groups at this early time point (Fig. 3c). There were additionally no differences in the hydraulic permeability between treatment groups as well (data not shown). Similarly, histological assessment showed similar cartilage repair between treatment groups 4 weeks post-surgery. Faint outlines of the original MST holes were present in both SmartShot^®^ conditions, whereas more prominent void spaces were visible in the K-wire group (Fig. 4a). Blinded ICRS II scoring revealed improved basal integration in the 0.9 mm SmartShot^®^ group compared to the microfracture group (Fig. 4b). There were no significant differences between treatment groups in the nine other parameters scored: defect fill, integration to surrounding cartilage, matrix staining, surface architecture, subchondral bone abnormality, vascularisation, surface/superficial assessment, mid/deep zone assessment and overall assessment. As evident from the representative SafO/FG images, matrix staining was minimal across all groups.

### Fluorochrome imaging

**Fig 5.**
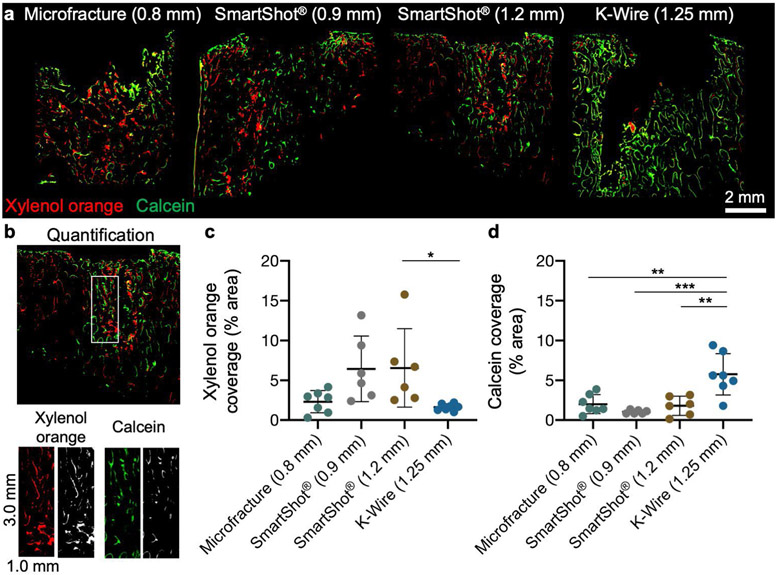
Bone remodelling over 4 weeks after MST. (**a**) Representative fluorochrome images at the midplane of the defects. Red: xylenol orange, injected at the time of surgery. Green: calcein, injected 2 weeks post-surgery. (**b**) Quantification of fluorochrome images. A 3.0 mm by 1.0 mm rectangular region nearest to the MST site was separated into the respective red and green channels and quantified. (**c,d**) Percentage area of xylenol orange and calcein, respectively. * *p* < 0.05, ** *p* < 0.01, *** *p* < 0.001. Bars indicate mean ± standard deviation. *n* = 6-7 holes/treatment. *N* = 6 animals.

Fluorochrome label incorporation at the time of surgery (xylenol orange) and 2 weeks post-surgery (calcein) showed marked differences in the bony remodelling response for each MST technique (Fig. 5a). For this analysis, a region of interest was set adjacent to the MST hole (Fig. 5b). The percent area of each mineral label was quantified within this region of interest. The defects treated with the SmartShot^®^ devices had the highest xylenol orange incorporation, indicative of early bone formation, while a delayed response was seen in the K-wire group (Fig. 5c). At the later time point (2 weeks), the K-wire group had significantly more calcein incorporation in comparison to all other groups (Fig. 5d). The defects treated with microfracture awl had relatively even percentages of each label, signifying a steady, but relatively quiet, bony response over the 4 weeks testing period.

### Global bony response

**Fig 6.**
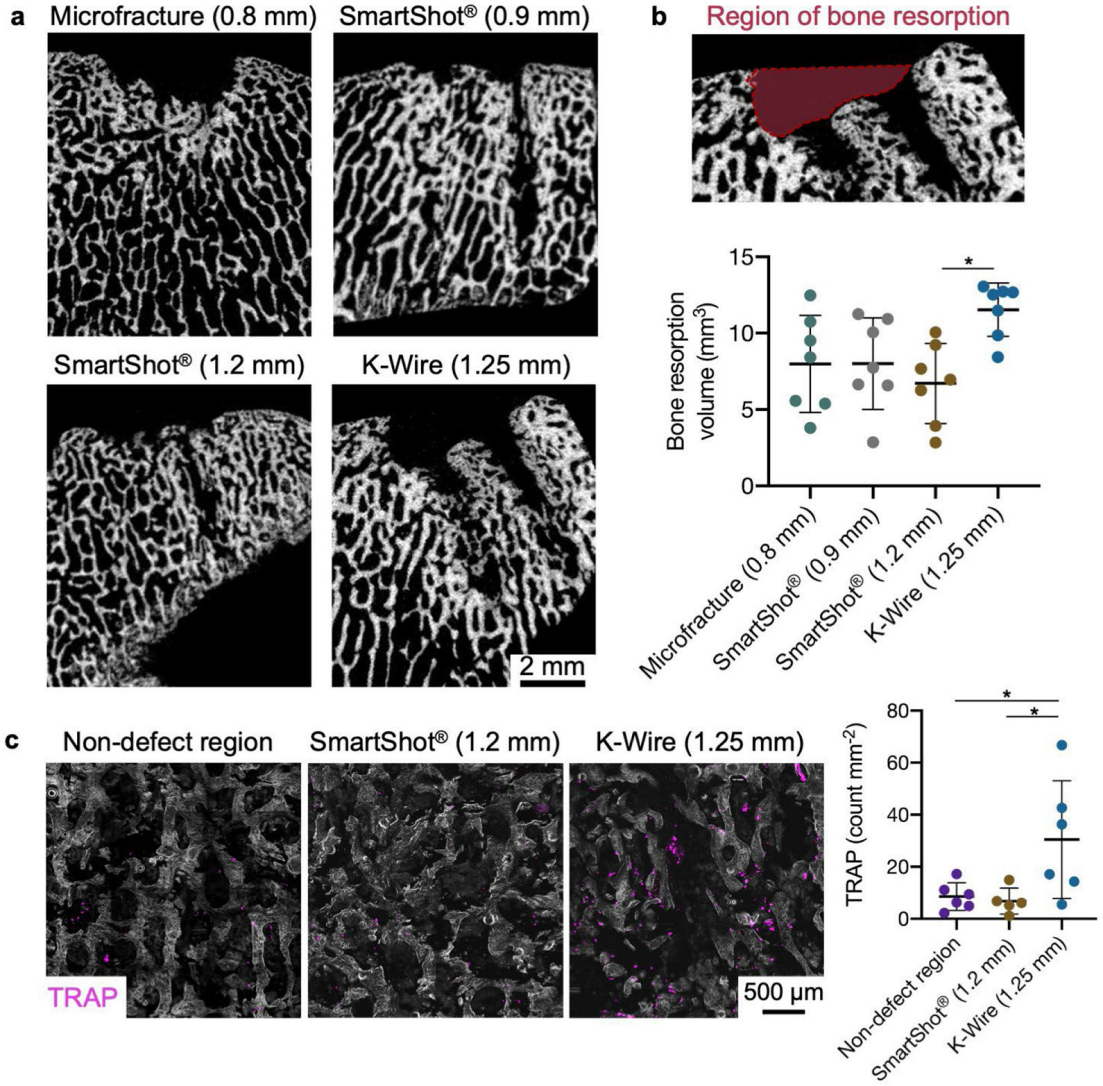
Global bony response underneath cartilage defects following MST. (**a**) Representative μCT images at the midplane of the defects. (**b**) 3D quantification of bone resorption. * *p* < 0.05. *n* = 7 defects/treatment. *N* = 6 animals. (**c**) TRAP staining and quantification. TRAP positive pixels were normalised to the bone area in a 7 mm by 7 mm image. * *p* < 0.05. Bars indicate mean ± standard deviation. *n* = 5-6 defects/treatment. *N* = 6 animals.

μCT was used to assess bone resorption in the subchondral bone underlying the cartilage defects 4 weeks post-surgery (Fig. 6a). Similar to the histology findings, the MST holes were most apparent in the K-wire group. For each treatment group, the MST holes were excluded from the bone resorption measurements. 3D quantification of bone resorption indicated that the K-wire group had significantly more bone resorption compared to the 1.2 mm SmartShot^®^. Cryosections stained for TRAP, which is expressed by osteoclasts, revealed that there was more osteoclastic activity in the subchondral region of defects treated with the K-wire in comparison to both the 1.2 mm SmartShot^®^ and native bone (Fig. 6b).

### Bone compaction

**Fig 7.**
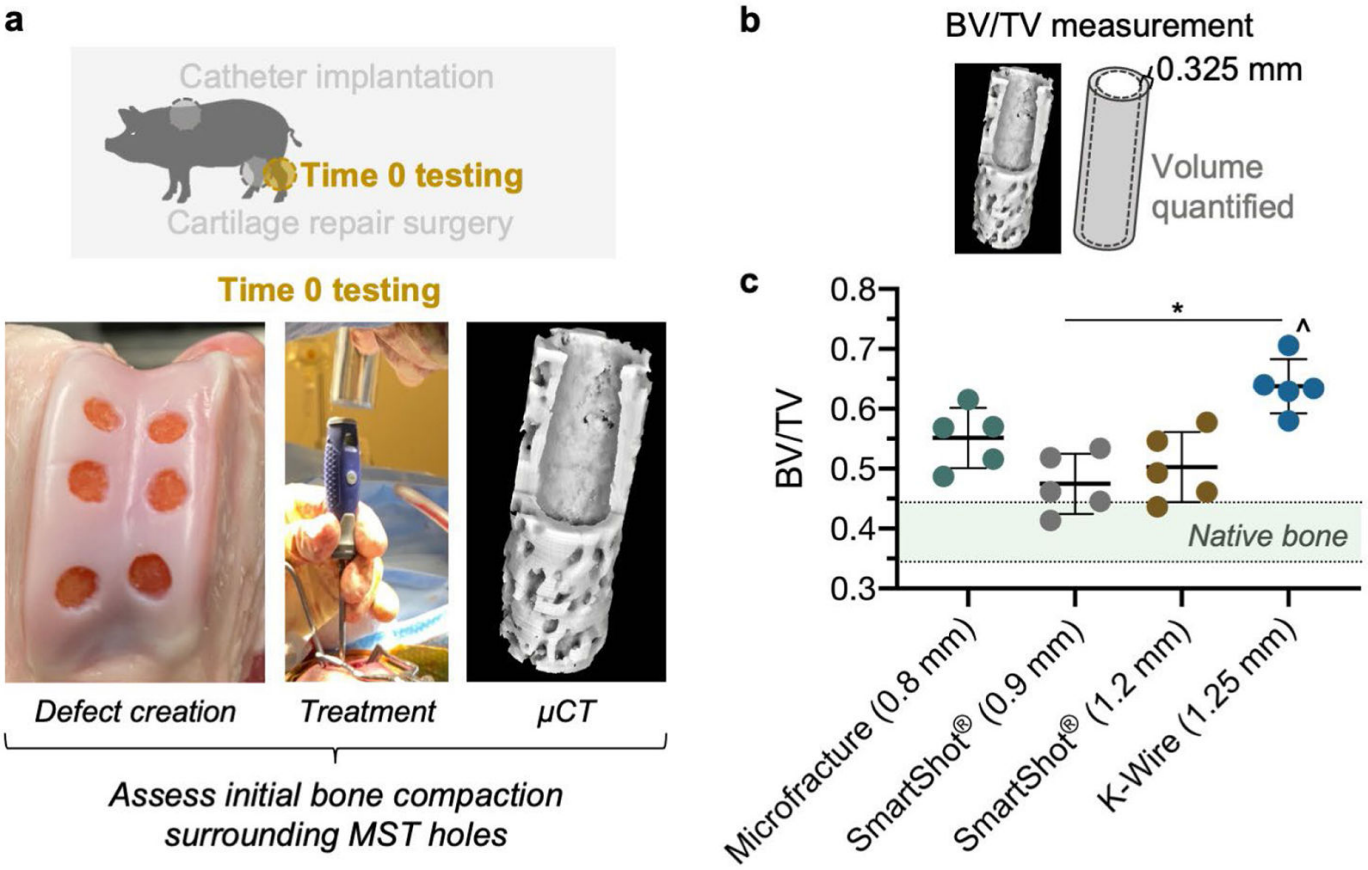
Bone compaction surrounding fresh (time 0) MST hole. (**a**) Schematic of time 0 testing. (**b**) BV/TV in region surrounding an MST hole. (**c**) Native bone was measured underneath a cartilage defect without any MST hole and the range of values are displayed by the shaded region. * *p* < 0.05. * *p* < 0.001 in comparison to native bone. Bars indicate mean ± standard deviation. *N* = 5 holes/treatment. *N* = 4 animals.

Immediately after euthanasia, the contralateral hind limbs were used to investigate the initial bone compaction caused by each MST device (Fig. 7a). For this time 0 assessment, chondral defects were created, treatment groups were randomised across defect locations and μCT imaging was performed on the osteochondral units after marrow access. Higher BV/TV measurements represented increased bone compaction. The K-wire group showed a significantly higher BV/TV in the region immediately adjacent to the marrow access holes in comparison to both the 0.9 mm SmartShot^®^ device and native bone. The range of BV/TV values measured from each SmartShot^®^ device overlapped with the native bone BV/TV range, unlike the microfracture and K-wire groups.

## Discussion

The present study directly compared bony changes that occurred post-MST using three different methods of marrow access hole creation: awl-based microfracture, needle puncture (SmartShot^®^) and subchondral drilling (using a K-wire). Although not expected to show differences, the study also characterised the cartilage repair quality from each of these treatments at the 4 week terminal time point. The histological appearance of the repair tissue was similar between treatments, apart from the 0.9 mm SmartShot^®^ enhancing basal integration (integration between the underlying bone and cartilage repair tissue) in comparison to the microfracture group. Comprehensive analyses were performed to assess bone quality post-MST including, histology, μCT and fluorochrome labelling. Taken together, these results highlighted potential disadvantages in the use of K-wires for subchondral drilling. The 1.25 mm K-wire caused the most bone resorption, delayed bony healing and compacted the bone around the perimeter of the MST holes in comparison to other marrow access methods. It may be that a fluted drill bit (Gao et al., 2018), designed to excise bone chips as the drill advances, would better preserve the subchondral bone in comparison to the smooth K-wire, however that was not assessed in the present study. A smaller diameter K-wire may have also caused less damage to the bone. Regardless, the 1.25 mm K-wires used in this study are within the range commonly used in clinical practice. As an alternative approach, the SmartShot^®^ device best preserved the subchondral bone. Interestingly, across all outcome measures, no statistical differences were found between the different sized SmartShot^®^ devices, suggesting that the method of hole creation may play a larger role than hole size in terms of the post-MST bony response.

It is important to consider not only the hole diameter and puncture method but also the hole depth and number. All of these variables likely play a role in the subsequent bony healing and cartilage repair. In the present study, the microfracture awl had the shortest hole depth (2.0 mm) and the SmartShot^®^ devices had the longest hole depth (8.0 mm), with the K-wire in the middle (6.0 mm hole depth). Because the two SmartShot^®^ devices had similar hole depths and identical methods of puncture, it was possible to more easily draw comparisons between these treatments. However, it should be noted that, given space constraints, 2 MST holes were created using the 1.2 mm diameter SmartShot^®^ and 3 MST were created using the 0.9 mm SmartShot^®^. This difference in hole number may have led these conditions to be more similar across the tested outcomes.

Previous studies have highlighted the variability in MST, from the technique itself (Kroell et al., 2016; Theodoropoulos et al., 2012) to patient outcomes (Knutsen et al., 2007; Mithoefer et al., 2009). There is considerable intra- and inter-surgeon variability with MST (Kroell et al., 2016), which likely contributes to the variable cartilage fill grade (18 to 95 %) (Mithoefer et al., 2009). While the focus of the present study was not on improving the repeatability of MST, results did support the use of the SmartShot^®^ (or a similar device), which could offer greater control and uniformity of hole depth across surgeons. Further, the use of this device in both the preclinical and clinical procedures may enhance the repeatability of outcomes between research groups and surgical teams.

One of the primary advances of this study was the implementation of fluorochrome labelling in a large animal. The present is the first study that used fluorochrome imaging to monitor the dynamic bone remodelling that occurred after MST techniques. This longitudinal outcome measure reduces the number of animals required to visualise bony changes over time. While others have performed fluorochrome labelling in minipigs (Funk et al., 2009), this technique is relatively uncommon in large animals. The scarcity of large-animal studies utilising fluorochrome labelling may be due to the fact that while such mineral labels can be injected subcutaneously into small animals, large animals require intravenous administration (van Gaalen et al., 2010). Additionally, many of the fluorochrome labels used in rodents are not compatible with larger animals. Specifically, alizarine complexone can lead to sudden death in minipigs (Ruehe et al., 2008). To ensure animal safety over the duration of the study, periodic blood samples were taken from the intravenous catheter and the blood samples were analysed for ionised calcium. While there were statistically significant differences in ionised calcium levels after injecting xylenol orange, these transient changes were likely not physiologically significant (that is, ionised calcium remained in the physiological range). Overall, the positive response of the animals to the described fluorochrome labelling protocol should encourage other groups to implement this potentially impactful outcome measure in studies of MST and osteochondral regeneration.

All large-animal studies are limited by sample size; nonetheless, an efficient utilisation of animals was demonstrated and significant differences were identified between treatment groups within the cohort, demonstrating the utility of study design to reduce animal numbers. Both the operative and non-operative hind limbs were used to establish two time points (time 0 and 4 weeks) for separate outcome measures. Additionally, both the cartilage repair quality and changes to the subchondral bone were assessed. While no differences in cartilage repair were expected at the 4 week time point (given how short the time period was), it was important to perform such assays to provide a baseline and motivation for future long-term studies evaluating next generation cell and material adjuvants to MST. Future work will also determine whether bone quality relates to repair tissue strength and durability.

## Conclusions

This study was built on previous literature suggesting that small diameter and deeper MST holes may reduce subchondral bone abnormalities and ultimately improve cartilage repair. While a few studies have compared tool sizing for MST, none have jointly investigated tool sizing and the method of hole creation. This study provided strong evidence in favour of a repeatable needle-puncture device (SmartShot^®^ or a similar device) to best preserve the subchondral bone and minimise aberrant remodelling after MST. This work also provided a cautionary tale regarding the use of K-wires, as this treatment group consistently performed poorly with respect to subchondral bone remodelling and healing. Overall, the results of the study should inform the design of future preclinical studies and has the potential to change current surgical practice.

## List of Abbreviations

AMIC: autologous matrix-induced chondrogenesis
ANOVA: analysis of variance
BV/TV: bone volume/total volume
IACUC: Institutional Animal Care and Use Committee
ICRS: International Cartilage Repair Society
K-wire: Kirschner wire
MST: marrow stimulation
OA: osteoarthritis
OCT: optimal cutting temperature
PBS: phosphate-buffered saline
PMMA: polymethyl methacrylate
SafO/FG: safranin O/fast green
TRAP: tartrate-resistant phosphatase
μCT: micro-computed tomography
